# The impact of climate-adaptive city construction pilot policies on corporate ESG performance

**DOI:** 10.3389/fpubh.2025.1646269

**Published:** 2025-09-15

**Authors:** Chen Cao, Xintong Zhang, Guilan Yu

**Affiliations:** ^1^School of Business and Management, Jilin University, Changchun, China; ^2^China Statistical Information Service Center (Public Opinion Survey Center of NBS), Beijing, China

**Keywords:** ESG, difference-in-differences, climate-resilient city, text mining, policy analysis

## Abstract

**Introduction:**

In the context of climate-adaptive urban construction policies, understanding their impact on corporate sustainability practices is of critical importance. This study investigates how such policies influence corporate Environmental, Social, and Governance (ESG) performance.

**Methods:**

A multi-period difference-in-differences (DID) approach combined with text mining techniques was employed to evaluate the effects of climate-resilient city construction policies on corporate ESG performance. To ensure robustness, additional analyses were conducted using Propensity Score Matching-Difference-in-Differences (PSM-DID), instrumental variable methods, and various robustness checks.

**Results:**

The findings demonstrate that climate-resilient city construction policies significantly improve corporate ESG scores, thereby promoting sustainable development practices within companies. Mechanism analysis further reveals that managerial environmental awareness serves as a critical mediating factor, suggesting that the policies indirectly enhance ESG performance by increasing managers’ focus on environmental issues.

**Discussion:**

This study provides empirical evidence that climate-adaptive urban construction policies contribute to improved corporate ESG outcomes. The results offer valuable insights for policymakers and practitioners, serving as a reference for the implementation of climate-resilient policies and for future theoretical and applied research on ESG performance.

## 1 Introduction

Under the backdrop of global climate warming and resource depletion, social and economic development faces unprecedented challenges. Climate change has led to an increase in extreme weather events, posing significant threats to urban infrastructure, residents’ lives, and economic operations (1). In response, an increasing number of countries and cities have developed and implemented climate adaptation policies to enhance urban resilience and sustainability (2, 3). Since the 18th National Congress of the Communist Party of China, China has introduced a series of policies to address climate change, specifically emphasizing the construction of “climate-adaptive cities” to safeguard urban competitiveness and improve residents’ well-being (4). Meanwhile, enterprises, as key participants in climate governance, bear indispensable responsibilities in this process. With growing societal attention to corporate accountability, Environmental, Social, and Governance (ESG) performance has become a crucial metric for assessing an enterprise’s sustainable development capacity (5). ESG not only reflects a company’s commitments to environmental protection, social contributions, and corporate governance but also directly impacts its recognition in capital markets and long-term competitiveness (6). In the context of sustainable development, enterprises are shifting their focus from merely maximizing profits to achieving comprehensive development in both financial and non-financial domains (7). However, the economic externalities of climate change cannot be effectively addressed solely through market mechanisms or corporate self-regulation (8). This necessitates the active involvement of enterprises in building climate-adaptive cities to leverage policy and resource support, achieve sustainable transformation, and improve their ESG performance. Thus, examining the impact of climate-adaptive city construction on corporate ESG performance and its underlying mechanisms holds significant theoretical and practical value. This research can also provide scientific evidence for policymakers and corporate managers in their decision-making processes.

Currently, research on climate-adaptive city construction primarily focuses on its macro-level impacts. Studies have found that climate-adaptive city initiatives enhance urban resilience (4, 9), improve cities’ capacity to adapt to climate change (4), and promote the ecological transformation of urban industries (10). These efforts reduce the risk of climate change-induced disasters, improve urban livability, and elevate sustainability levels (11). Additionally, such initiatives foster innovation in climate-related financing, boost green industries, and drive climate-focused economic development (12). The construction of climate-adaptive cities aligns with people’s aspirations for a better life (13), contributes to the establishment of regional resilient city demonstration zones, and complements smart city development by forming mutually reinforcing effects (14). However, research at the micro level regarding climate-adaptive city construction remains limited. Among the few studies available, findings primarily indicate that such initiatives encourage enterprises to fulfill their environmental responsibilities (15).

Although early studies have provided valuable insights into the field of climate-adaptive city construction, two significant gaps remain. First, the research perspective is relatively narrow. Existing studies on climate policies largely focus on their macro-level impacts (4, 9), with limited attention paid to their micro-level effects, particularly on corporate ESG performance. Even among the few studies addressing corporate ESG performance, the scope of discussion is often limited to environmental responsibilities (15), lacking a comprehensive exploration of the social and governance dimensions. This lack of research on the micro-level impacts of climate policies on corporate ESG performance leaves critical issues insufficiently examined. Specifically, it hinders our understanding of how policies concretely influence corporate practices in environmental, social, and governance areas. Moreover, it prevents policymakers from accurately evaluating the real impact of policies at the corporate level, which could compromise the precision of policy design and its effectiveness in implementation. To address this gap, this study aims to explore the comprehensive impact of climate-adaptive city construction on corporate ESG performance, going beyond the singular dimension of environmental responsibility. Thus, investigating the effects of climate-adaptive city construction on corporate ESG performance constitutes the first objective of this research.

The second limitation in existing literature lies in the constrained exploration of the mechanisms underlying the impact of climate-adaptive city construction. Among the limited studies addressing corporate responsibility, green innovation has been considered as a mediating variable (15). However, this approach has notable shortcomings in explaining the policy transmission mechanism. First, green innovation primarily focuses on the development of environmentally friendly technologies and products at the corporate level, emphasizing specific innovation pathways. It fails to capture how policies indirectly influence corporate behavior through internal cognitive and decision-making processes. Second, prior research has overlooked the critical role of management in the policy transmission process, resulting in an incomplete understanding of the pathways through which policies affect corporate behavior. Third, green innovation as a mediating variable leans toward a technological implementation pathway, which is often constrained by industry characteristics and technological conditions, limiting its applicability across different contexts. Finally, green innovation tends to emphasize technical and product innovation while exerting relatively weak influence on fostering deep-seated environmental awareness and long-term environmental responsibility culture within organizations. To address these limitations, this study introduces managerial environmental attention as a mediating variable, providing a more comprehensive explanation of the policy transmission mechanism. This approach seeks to complement the inadequacies of green innovation by focusing on the role of management in shaping corporate responses to climate policies. First, managerial environmental attention—focusing on the top executives’ awareness and cognition of environmental issues—can reveal how policies indirectly influence corporate ESG performance through the value orientation and priorities of management. Compared to green innovation, this psychological variable not only clarifies the policy transmission mechanism but also enables quicker corporate responses to policy changes, allowing firms to flexibly adjust resource allocation and strategies. Additionally, the level of managerial awareness and attention to environmental issues directly determines the strategic priorities of a company in terms of environmental, social, and governance dimensions. When executives place high importance on environmental issues, they are more likely to integrate environmental responsibility into the company’s core strategy, allocate resources, and establish budgets to support sustainable development. Moreover, managerial environmental attention has broader applicability. It is relevant across companies of varying sizes, industries, and developmental stages, thereby enhancing the generalizability of research findings. Finally, the environmental focus of management also influences the internal corporate culture, fostering greater environmental awareness among employees and encouraging active identification with environmental goals. This mediating mechanism helps to open the “black box” between climate policies and corporate behavior, providing a more complete explanation of how policies impact corporate environmental performance from a psychological perspective. This comprehensive framework not only enriches the understanding of policy impact pathways but also offers valuable insights for future policymaking and corporate environmental management strategies.

To address these gaps, this study investigates the comprehensive impact of climate-adaptive city construction on corporate ESG performance and introduces managerial environmental attention as a mediating variable in the transmission mechanism. By doing so, it not only addresses the micro-level and mechanism-related shortcomings of previous research but also further elucidates how policies influence corporate environmental, social, and governance (ESG) practices through the cognition and attention of top management. This approach contributes to more precise policymaking, enhances corporate proactivity in participating in climate-adaptive city construction, and promotes sustainable corporate development. Furthermore, the findings provide scientific evidence and practical insights for optimizing future policies and improving corporate management practices. In addition to the above points, this study introduces methodological innovations. Specifically, it employs advanced text mining techniques to measure the mechanism variables. Using cutting-edge text analysis methods, the study leverages Python to analyze the frequency of environmental protection-related terms in the “Management Discussion and Analysis” section of corporate annual reports as a proxy for the environmental attention of executive teams.

In summary, this study explores how firms respond to climate-resilient city construction policies through changes in their ESG performance, and makes the following three theoretical contributions:

First, unlike existing literature that mainly focuses on traditional coercive environmental policies, such as carbon trading, environmental taxes, and emission constraints, this study focuses on the “climate-resilient city construction” policy, which is a novel policy tool characterized by cross-departmental coordination, non-coercive features, and planning orientation. This expands the research boundaries regarding the influence of climate policies on corporate behavior. Such policies are more indirect in institutional arrangements, and their impact pathways are more complex. The micro-level impact mechanisms of these policies on corporate sustainability behavior have not been fully explored in existing studies.

Second, this study introduces “managerial environmental attention” as a mediating mechanism, constructing a cognitive transmission path from the perspective of managerial cognition and attention allocation. This approach breaks away from previous studies’ reliance on material mechanisms, such as “green innovation” or “resource investment.” It emphasizes how policies, through shaping managerial value orientation, guide firms to respond to sustainability issues at the strategic and cultural levels. This approach is particularly applicable to China’s “top-down + incentive-driven” climate adaptation governance system. In doing so, it indirectly moderates corporate behavior and enriches the theoretical understanding of how policies influence corporate actions from an organizational cognition perspective.

Third, the study integrates and deepens multiple theoretical perspectives. By combining stakeholder theory with the attention-based view of the firm, it explains how external institutional pressures can reshape managerial focus and influence corporate decision-making. This integrated framework enhances the theoretical coherence and explanatory power of the study.

Therefore, this study is not only innovative in its research objects and policy types but also extends and deepens the theoretical pathways of explanation, offering a new perspective for understanding how structural climate policies influence corporate ESG behavior through cognitive mechanisms.

## 2 Theoretical analysis and research hypotheses

### 2.1 The promotive effect of climate-adaptive city construction on corporate ESG performance

Based on stakeholder theory, the sustainable development of enterprises relies not only on shareholder interests but also on addressing the needs of other stakeholders, including responsibilities in the environmental, social, and governance (ESG) dimensions (16) Policies serve as external governance mechanisms for corporate environmental responsibility, constraining managerial behavior by enhancing information disclosure and transparency (17). Climate-adaptive city construction, as a systemic policy involving governments, enterprises, communities, and the environment, has a profound impact on corporate ESG performance. First, in the environmental dimension, climate policies promote green infrastructure, low-carbon buildings, and clean energy usage, enhancing urban climate resilience and reducing resource dependency (18). This policy orientation creates an external environment conducive to low-carbon development, enabling enterprises to access resources and policy support for emission reduction and energy efficiency, thereby lowering their carbon footprint. Environmental oversight policies, such as environmental protection inspections, strengthen government regulation and improve corporate ESG performance. For instance, the implementation of carbon emissions trading pilots has been shown to enhance the quality of ESG information disclosure (19) and improve overall ESG performance (20). According to the Porter Hypothesis (8), while environmental regulations may increase compliance costs, they can also stimulate technological innovation, resulting in economic compensation through energy savings, product quality improvements, and production efficiency gains, thus achieving a win-win for economic performance and ESG outcomes (21). Second, in the social dimension, climate-adaptive cities prioritize not only environmental protection but also public health, community well-being, and social equity. These social responsibility goals drive improvements in corporate performance in the social dimension (22). According to stakeholder theory, climate policies increase scrutiny from investors, media, and customers regarding corporate environmental performance, thereby raising corporate social responsibility awareness. With stricter environmental regulations, companies are more inclined to improve their ESG performance, enhancing their social responsibility efforts (23). Furthermore, corporate social responsibility actions in addressing climate change significantly enhance their social influence and corporate image (24). Thus, the social responsibility demands arising from climate-adaptive city construction promote corporate ESG performance in the social dimension. Finally, in the governance dimension, climate policies typically include multi-stakeholder oversight and governance requirements, imposing higher standards for corporate transparency and compliance (25). These policies effectively constrain managerial self-serving behaviors, reducing their potential harm to ESG performance (26). Additionally, the implementation of environmental policies drives companies to strengthen their internal governance structures to better respond to policy pressures and market demands (6). Strict environmental regulatory oversight contributes to improved internal control systems, thereby enhancing corporate ESG performance in the governance dimension (27).

As a guiding and systemic national strategy, the Climate-Resilient City Construction policy does not impose direct and mandatory regulatory requirements on firms. However, in practice, its implementation at the local government level—through mechanisms such as planning review, project certification, and performance evaluation—significantly strengthens the institutional pressure faced by enterprises in the regions covered by the policy.

Drawing on stakeholder theory, we argue that the policy exerts indirect constraints on firms by reshaping the structure of stakeholder influence. First, governmental pressure arises as local governments incorporate green performance indicators into official assessments, transmitting environmental requirements to the corporate level through administrative channels. Second, capital market pressure emerges as investors reassess firms’ ESG-related risks; when policy signals a strong commitment to sustainability, firms that respond slowly may face valuation discounts or financing constraints. Third, public and societal pressure stems from heightened scrutiny by the media, the general public, and environmental NGOs, forming external reputational incentives and even accountability risks.

These mechanisms collectively illustrate how the policy indirectly governs corporate behavior by restructuring the stakeholder environment in which firms operate, thereby creating institutional momentum for strategic response at the managerial level.

In summary, based on stakeholder theory, climate-adaptive city construction promotes corporate ESG performance by encouraging responsibility across environmental, social, and governance dimensions. Through policy pressure, resource support, and stakeholder attention, companies not only enhance their sustainable competitiveness but also contribute positively to the development of a low-carbon, inclusive, and transparent societal environment. This study provides systematic theoretical and empirical support for understanding the mechanisms through which climate-adaptive city construction influences corporate ESG performance. Based on the above, this study proposes the following hypothesis:

*H1*: Climate-adaptive city construction positively promotes corporate ESG performance.

### 2.2 The mediating role of managerial environmental awareness

Climate-related policies influence not only the external operating environment of enterprises through policy guidance and resource allocation but also indirectly enhance managerial attention to environmental issues, thereby further affecting corporate ESG performance (28). Managerial environmental awareness refers to the degree of importance and recognition that top executives assign to environmental issues. This attention effectively steers enterprises toward prioritizing environmental, social, and governance (ESG) factors in resource allocation, strategy formulation, and daily operations (29). The implementation of climate-adaptive city policies is often accompanied by higher environmental standards, creating external pressures that compel managers to increase their focus on environmental issues and strengthen their sense of environmental responsibility (30). In responding to external climate policy pressures, managers are inclined to integrate environmental priorities into the core corporate strategy, aiming to enhance ESG performance to meet increasingly stringent regulatory requirements (31). Furthermore, under intense external environmental pressures, managerial attention tends to prioritize corporate environmental behavior, fostering a heightened awareness of ESG within the organization (32). This finding suggests that managerial environmental attention is not merely a superficial response to policy pressure but also contributes to the establishment of a long-term ESG culture within the company.

Managerial environmental awareness also plays a critical role in resource allocation. For instance, a high level of environmental attention among management is significantly associated with increased investment in sustainable development technologies, a tendency particularly evident under the context of climate-adaptive policies (29). Managers with heightened environmental awareness are more willing to invest in projects that enhance environmental performance, thereby strengthening the company’s recognition in capital markets and bolstering long-term competitiveness (33). The policy requirements brought by climate-adaptive city construction further reinforce managerial preferences for environmentally oriented resource allocation. This is reflected in multi-dimensional improvements in corporate ESG performance through green investments, risk management, and enhanced transparency. Beyond resource allocation, managerial environmental awareness also positively influences corporate culture and employee behavior. When management prioritizes environmental issues, it raises environmental awareness among employees, fostering a unified culture of sustainability within the organization—an essential factor for effective corporate social responsibility practices (34). Under the external pressures of climate-adaptive city construction, managerial environmental awareness serves to transmit external policy signals into the organization. By shaping a proactive ESG culture, management can drive the company toward long-term sustainable development. As a key link between external policies and internal corporate behavior, managerial environmental attention establishes a crucial transmission pathway between policy requirements and corporate responses.

Although the Climate-Resilient City Construction policy does not impose direct regulatory constraints on firms, as a systemic policy it reshapes the external stakeholder environment, indirectly influencing corporate sustainable behavior.

Based on the Attention-Based View (47), the allocation of attention by top executives plays a central role in shaping organizational strategy, as it determines how resources are distributed and which issues are prioritized. In this context, the institutional pressure and external uncertainty triggered by policy implementation act as key forces that reconfigure managerial cognitive priorities.

Specifically, on the one hand, institutional arrangements associated with the policy—such as mandatory environmental disclosure, performance evaluations, and green access thresholds—compel managers to increase their attention to environmental issues in order to avoid compliance and reputational risks. On the other hand, the policy generates resource-based incentives, such as green financing channels, government project support, and tax benefits, which heighten managers’ resource sensitivity toward environmental strategies.

Taken together, these changes in the institutional and resource environment prompt corporate executives to focus more intensively on sustainability concerns, thereby driving the formulation and implementation of ESG-related actions.

In summary, managerial environmental awareness plays a critical mediating role in the relationship between climate-adaptive city construction and corporate ESG performance. It not only enables enterprises to better respond to external policy pressures but also drives improvements in environmental, social, and governance dimensions by prioritizing resource allocation and fostering internal cultural development. This mediating mechanism provides a more comprehensive perspective on how climate-adaptive policies specifically influence corporate ESG, offering valuable insights for policymakers to design more effective transmission mechanisms for promoting corporate sustainability. Accordingly, the following hypothesis is proposed:

*H2*: Managerial environmental awareness mediates the relationship between climate-adaptive city construction and corporate ESG performance, whereby climate-adaptive city construction influences corporate ESG performance through managerial environmental awareness.

## 3 Research design

### 3.1 Research sample and data sources

This study selects A-share listed companies on the Shanghai and Shenzhen Stock Exchanges in China from 2010 to 2021 as the initial sample. The sample is screened as follows: First, data from ST and *ST companies are excluded. Second, observations with missing values for key variables during the sample period are removed. Additionally, to mitigate the influence of outliers in the empirical analysis, the main continuous variables are winsorized at the 1 and 99% levels. Data on executive and corporate financial characteristics are obtained from the CSMAR and Wind databases, with cross-verification performed. Corporate annual report data are sourced from the CNINFO website and processed using custom programming. All other data are derived from the CSMAR database and the Chinese Research Data Services Platform (CNRDS).

### 3.2 Variable definitions

#### 3.2.1 Dependent variable

Corporate ESG Performance (ESG): This study adopts the Huazheng ESG Rating system data to measure corporate ESG performance, following the methodologies of Wang et al. (35) and Hu and Wang (36). After comparing data from multiple ESG rating agencies, we found that the Huazheng ESG rating system is the most suitable for this study. First, Huazheng’s evaluation framework is grounded in core ESG principles and international best practices, while being tailored to the Chinese market context. It adopts a transparent, top-down three-tier indicator system, consisting of 3 primary indicators, 14 secondary indicators, 26 tertiary indicators, and over 130 underlying data points. Second, the Huazheng system integrates both traditional financial data and alternative data sources through an AI-driven big data engine, which performs quarterly updates and dynamic tracking to systematically evaluate the ESG performance of all A-share listed companies over the past decade. Finally, due to its comprehensive framework and broad coverage, the Huazheng ESG system is widely regarded as one of the most authoritative ESG data sources for Chinese listed firms (35). Its extensive data availability significantly enhances the representativeness and validity of our sample.

#### 3.2.2 Independent variable

Climate-Adaptive City Construction Policy (Policy): This variable is constructed as the interaction term between the treatment group dummy variable (treat) and the policy timing dummy variable (time). The dummy variable treat is assigned a value of 1 for cities included in the climate-adaptive city construction policy and 0 otherwise. The dummy variable time is assigned a value of 1 for years after the implementation of the climate-adaptive city construction policy and 0 otherwise.

#### 3.2.3 Mechanism variable

Managerial Environmental Awareness (TTA): The core mechanism variable of this study is managerial environmental awareness (TTA). Following the approaches of Ding et al. (37) and Barrett et al. (38), this study measures managerial environmental attention based on the frequency of environment-related terms in the “Management Discussion and Analysis” (MD&A) sections of corporate annual reports. The specific construction process is as follows: First, using Python web scraping tools, annual reports of A-share listed companies from 2010 to 2021 are collected from the CNINFO website, and the MD&A sections are extracted. Second, based on the environmental vocabulary lists provided by Ding et al. (37) and Lv and Wang (39), relevant environmental terms are identified. Third, Python’s “Jieba” library is used for text segmentation, and the total word count of the MD&A sections is calculated. Finally, the frequency of environment-related terms is calculated as a proportion of the total word count, reflecting the level of managerial environmental awareness.

#### 3.2.4 Control variables

Following existing studies by Fu et al. (40) and Rauf et al. (41), this study includes the following control variables. To account for factors that may influence corporate ESG performance, the study incorporates a series of control variables, specifically: Return on Assets (ROA): The ratio of net profit to total assets; Leverage (Lev): The ratio of total liabilities to total assets; Ownership Concentration (Top 1): The shareholding ratio of the largest shareholder; Growth (Growth): Measured using asset growth rate, calculated as the annual increase in fixed assets relative to the initial value at the beginning of the period; Return on Equity (ROE): The ratio of net profit to average equity; Firm Age (FirmAge): The logarithm of the difference between the current year and the year of the firm’s establishment; Ownership Type (SOE): Assigned a value of 1 if the firm is state-owned, and 0 otherwise; Auditor Type (Big4): Assigned a value of 1 if the auditing firm is one of the “Big Four” accounting firms, and 0 otherwise.

### 3.3 Model construction

This study examines the impact of climate-adaptive city construction policies, which are implemented incrementally over multiple years. Unlike the traditional Difference-in-Differences (DID) method that is typically suited for assessing the effect of policies implemented at a single time point, the staggered nature of this policy results in changes in the treatment group across different implementation years. To account for the staggered timing of policy implementation across different cities, we employ a multi-period difference-in-differences (DID) methodology. In this framework, the treatment variable is constructed as an interaction term between a dummy variable for the treatment group (indicating cities that have implemented the climate-resilient city policy) and a time dummy reflecting the specific year when the policy was implemented in each city. This design ensures that each city enters the treatment group only after the actual launch of the policy in that city, capturing the temporal variation in the policy’s impact. Referring to related studies by Li et al. (42), Zhang et al. (43), and Enciso-Alfaro et al. (44), this study constructs the following staggered DID model:


$${\mathrm{ESG}}_{\mathrm{it}}=\beta_{0}+\beta_{1}{\text{Polocy}}_{\mathrm{it}}+\beta_{2}{\mathrm{Con}}_{\mathrm{it}}+\theta_{i}+\delta_{t}+\varepsilon_{\mathrm{it}}$$

Where:

${\mathrm{ESG}}_{\mathrm{it}}$ : Represents the ESG score of firm *i* in year *t*; ${\text{Polocy}}_{\mathrm{it}}$ : Represents the interaction term between the treatment group dummy variable (treat) and the policy timing dummy variable (time). treat equals 1 if city i is a climate-adaptive city pilot, and 0 otherwise. Time equals 1 if city i implemented the policy in year *t*, and 0 otherwise; *β*: Captures the effect of the climate-adaptive city construction policy on ESG performance; ${\mathrm{Con}}_{\mathrm{it}}$ : Denotes a vector of control variables; $\theta_{i}$ : Represents city fixed effects; $\delta_{t}$ : Represents year fixed effects; $\varepsilon_{\mathrm{it}}$ : Denotes the random error term. Unless otherwise specified, the empirical analyses in this study are estimated using robust standard errors clustered at the industry level.

## 4 Empirical analysis and discussion

### 4.1 Descriptive statistics

Based on the descriptive statistics in Table 1, the variables show varying degrees of distribution across firms, reflecting diversity in policy participation, environmental awareness, financial indicators, and governance characteristics. The mean ESG rating (ESG) is 6.45 with a standard deviation of 1.15, while the maximum and minimum values are 9.00 and 1.00, respectively, indicating significant differences in ESG performance among firms. For climate-adaptive city construction (Policy), the mean value is 0.21 with a standard deviation of 0.41, suggesting that while most firms are not extensively involved in the policy, some actively participate. Growth (Growth) has a mean of 0.18 and a standard deviation of 0.42, with large differences between the maximum and minimum values, reflecting substantial disparities in firm growth. Profitability measures such as return on assets (ROA) and return on equity (ROE) have mean values of 0.04 and 0.07, respectively, with relatively small standard deviations of 0.07 and 0.14, indicating less variation in these metrics across firms. Financial leverage (Lev) has a mean of 0.42 and a standard deviation of 0.21, showing notable differences in firms’ debt levels. Firm age (FirmAge) has a mean of 2.87 and a standard deviation of 0.36, suggesting a concentrated age distribution. The mean value of state-owned enterprises (SOE) is 0.33, with a standard deviation of 0.47, indicating that a portion of the sample consists of state-owned firms. Ownership concentration (Top1) has a mean of 0.34 and a standard deviation of 0.15, reflecting variation in ownership structures. Auditor type (Big4) shows a mean value of 0.06, indicating that only a small proportion of firms are audited by the “Big Four” accounting firms. The proximity to rivers (IV1) has a mean of 0.41 and a standard deviation of 0.34, indicating significant geographical differences. Following established literature, we use river proximity as an instrumental variable for the implementation of climate-resilient city construction policies. The rationale is that cities located closer to major river systems are more prone to climate-related risks such as flooding and thus are more likely to be selected as pilot regions for climate adaptation programs. However, river proximity is a geographic and time-invariant feature that does not directly affect firms’ ESG performance except through its influence on policy assignment. This approach is consistent with prior studies that use geographic features as instruments, such as Nunn and Qian (45), who employ the suitability for growing wheat and rice as instruments, and Bosker and Buringh (46), who use river proximity to study the emergence of cities. In our setting, river proximity satisfies the relevance condition by predicting policy assignment and the exclusion restriction by not directly affecting the dependent variable (ESG), conditional on controls and fixed effects.

**Table 1:** Descriptive statistics.

Variable name	Mean	Standard deviation	Maximum	Minimum
ESG	6.45	1.15	9.00	1.00
Policy	0.21	0.41	1.00	0.00
Growth	0.18	0.42	4.33	−0.66
ROA	0.04	0.07	0.25	−0.40
ROE	0.07	0.14	0.41	−1.07
Lev	0.42	0.21	0.92	0.03
FirmAge	2.87	0.36	3.61	1.10
SOE	0.33	0.47	1.00	0.00
Top1	0.34	0.15	0.76	0.08
Big4	0.06	0.23	1.00	0.00
IV1	0.41	0.34	1.40	0.00
TTA	0.05	0.04	0.19	0.00

The lagged climate-adaptive city construction policy (lagPolicy) has a mean of 0.18 and a standard deviation of 0.39, reflecting differences in policy implementation across firms. Managerial environmental awareness (TTA) has a mean of 0.05 and a standard deviation of 0.04, indicating that managerial attention to environmental issues is generally low and concentrated. Overall, the variables exhibit noticeable distributional differences across firms, reflecting a diversity and imbalance in policy participation, environmental awareness, financial metrics, and governance characteristics.

### 4.2 Baseline regression

Table 2 presents the baseline regression results of the impact of climate-adaptive city construction policies on corporate ESG ratings. Columns (1) and (2) respectively show the effects of this policy on corporate ESG ratings. In Column (1), the coefficient of the climate-adaptive city construction policy is 0.577, significant at the 10% level, indicating a positive effect of the policy on ESG ratings. In Column (2), the policy coefficient is 0.096, significant at the 1% level, providing further evidence of the positive impact of the policy in enhancing ESG ratings. These results suggest that the climate-adaptive city construction policy significantly improves corporate ESG ratings. Additionally, Column (2) includes more control variables, revealing the following insights: return on assets (ROA), return on equity (ROE), the largest shareholder’s ownership ratio (Top1), and auditing by the “Big Four” (Big4) all have significant positive impacts on ESG ratings, with coefficients of 0.736, 0.221, 0.536, and 0.138, respectively, significant at various levels.

**Table 2:** Baseline regression.

	ESG (1)	ESG (2)
Policy	0.169*** (0.035)	0.096*** (0.018)
Growth		−0.016 (0.011)
ROA		0.736*** (0.190)
ROE		0.221** (0.038)
Lev		−0.354*** (0.045)
FirmAge		−0.174** (0.065)
SOE		0.027 (0.032)
Top1		0.536*** (0.076)
Big4		0.138*** (0.044)
*R* ^2^	0.597	0.625
Industry fixed effects	Yes	Yes
Year fixed effects	Yes	Yes
Sample size	31,978	31,978
Cluster	Yes	Yes

** and *** indicate significance at the 5% and 1% levels, respectively; values in parentheses are standard errors.

These findings indicate that higher profitability and better corporate governance quality contribute to improved ESG ratings. In contrast, leverage (Lev) and firm age (FirmAge) have significant negative impacts on ESG ratings, with coefficients of −0.354 and −0.174, respectively, significant at higher levels. This suggests that higher leverage and longer firm history may hinder improvements in ESG ratings. These negative effects highlight the importance of considering factors such as capital structure and company age when formulating corporate sustainability strategies to mitigate potential adverse impacts on ESG performance. Overall, the positive impact of the climate-adaptive city construction policy on corporate ESG ratings is robust. Meanwhile, financial and governance factors also play significant roles in influencing ESG ratings. These findings demonstrate the practical effectiveness of the policy in promoting corporate sustainability. Hypothesis H1 is supported, confirming that climate-adaptive city construction policies have a significant positive effect on corporate ESG performance. These results provide empirical evidence for the effectiveness of such policies in enhancing corporate social responsibility performance.

### 4.3 Parallel trend test

The parallel trend test suggests that before the implementation of the pilot, the treatment and control groups exhibited similar trends in Huazheng ESG scores, satisfying the parallel trend assumption. After the pilot began, the estimated coefficients show a significant positive effect, which becomes more pronounced and uncertain in subsequent years. This reflects the gradually increasing and strengthening positive impact of the pilot on Huazheng ESG scores.

### 4.4 Endogeneity test

#### 4.4.1 PSM-DID method

Supplementary Table S1 presents the results of addressing endogeneity using the propensity score matching (PSM) combined with the difference-in-differences (DID) method, aimed at verifying the robustness of the causal relationship between climate-adaptive city construction and corporate ESG performance. The results show that the DID coefficient for climate-adaptive city construction is 0.097, significant at the 1% level (with a standard error of 0.023). This indicates that even after matching characteristics between samples, climate-adaptive city construction continues to have a significant positive impact on corporate ESG performance. Additionally, the R^2^ value of 0.680 demonstrates the model’s strong explanatory power for the dependent variable, with industry fixed effects and year fixed effects controlled. These findings suggest that after matching and controlling for various fixed effects, the positive impact of climate-adaptive city construction on corporate ESG performance remains robust, further ruling out biases caused by endogeneity issues. This reinforces the reliability and validity of the estimated results.

#### 4.4.2 Instrumental variable (IV) method

Table 3 presents the instrumental variable regression results for the climate-resilient city construction policy (Policy). Using two-stage least squares (2SLS) estimation, the findings indicate a significant positive effect of the policy on the outcome variable. In the first-stage regression, the instrumental variable “River IV1” is statistically significant at the 1% level, suggesting a strong correlation with the policy variable and thus providing a valid instrument for the second-stage estimation. The second-stage regression results show that the coefficient of the policy variable is 0.395 across different model specifications, all statistically significant at the 1% level, indicating that the climate-resilient city policy has a robust positive effect on the dependent variable. Control variables as well as industry and year fixed effects are included in the model to improve robustness. Furthermore, the Cragg-Donald Wald *F*-statistic and the Sargan overidentification test support the strength and validity of the instrument, mitigating concerns about weak instruments and confirming the exogeneity assumption. The Durbin–Wu–Hausman test also suggests that the policy variable is endogenous, thereby justifying the use of the instrumental variable approach. Taken together, these statistical tests consistently support the significant positive effect of the climate-resilient city construction policy and confirm the validity and robustness of the model.

**Table 3:** Instrumental variable regression results.

	(1) First-stage regression	(2) Second-stage regression
IV1	0.223*** (0.006)	
Policy		0.395*** (0.077)
Control variables	Yes	Yes
Industry fixed effects	Yes	Yes
Year fixed effects	Yes	Yes
Sample size	31,968	31,978
Cragg-Donald Wald *F*		1196.83***
Sargen		0.000
Durbin Wu–Hausman		15.62***

*** indicate significance at the 1% level, respectively; values in parentheses are standard errors.

### 4.5 Robustness tests

Table 4 presents the results of robustness tests, including checks using lagged dependent variables, combined fixed effects, and winsorization. In Column (1), the coefficient of the climate-adaptive city construction policy is 0.083, significant at the 1% level (standard error = 0.019), indicating a positive and significant impact of the policy on the lagged dependent variable. In Column (2), under the combined fixed effects, the policy coefficient is 0.106, also significant at the 1% level (standard error = 0.026), further validating the robustness of the results. In Column (3), with winsorized data, the policy coefficient remains significant at the 1% level with a value of 0.101 (standard error = 0.018), demonstrating that the policy’s positive effect on the dependent variable persists across different data processing methods. Overall, the three robustness tests consistently show that the climate-adaptive city construction policy maintains a positive and significant impact on the dependent variable under various methodological adjustments, confirming the robustness and consistency of the results.

**Table 4:** Robustness tests.

	Lagged dependent variable (1)	Combined fixed effects (2)	Winsorization (3)
Policy	0.083*** (0.019)	0.106*** (0.266)	0.101*** (0.018)
Control variables	Yes	Yes	Yes
*R* ^2^	0.662	0.650	0.630
Industry fixed effects	Yes	Yes	Yes
Year fixed effects	Yes	Yes	Yes
Industry-year combined Fixed effects	No	Yes	No
Sample size	28,840	31,978	31,920
Cluster	Yes	Yes	Yes

*** indicate significance at the 1% level, respectively; values in parentheses are standard errors.

### 4.6 Placebo test

The placebo test results show that most of the effect values are concentrated around zero, forming a typical normal distribution pattern without significant deviations. This indicates that in the absence of actual treatment, the randomly assigned “placebo” treatment effects do not significantly deviate from zero, thereby further supporting the validity of the DID model. In other words, no spurious treatment effects were detected, providing strong evidence for the robustness and reliability of the multi-period DID model employed in this study.

### 4.7 Further mechanism test

According to the mediation mechanism test results presented in Table 5, the impact of climate-adaptive city construction policies on managerial environmental attention and corporate ESG scores was analyzed. In Column (1), the climate-adaptive city construction policy has a significant positive effect on managerial environmental awareness, with a regression coefficient of 0.025, significant at the 1% level (*p* < 0.01). This indicates that the policy effectively increases managerial attention to environmental issues. In Column (2), the direct impact of the climate-adaptive city construction policy on corporate ESG scores is significant, with a regression coefficient of 0.081, significant at the 1% level (*p* < 0.01). Furthermore, managerial environmental attention significantly impacts corporate ESG scores, with a regression coefficient of 0.118, also significant at the 1% level (*p* < 0.01). This demonstrates that managerial environmental attention plays a mediating role between the policy and corporate ESG scores.

**Table 5:** Mediation mechanism test.

	TTA (1)	ESG (2)
Policy	0.025*** (0.018)	0.081*** (0.018)
TTA		0.118*** (0.015)
Control variables	Yes	Yes
*R* ^2^	0.647	0.629
Industry fixed effects	Yes	Yes
Year fixed effects	Yes	Yes
Sample size	31,908	31,908
Cluster	Yes	Yes

*** indicate significance at the 1% level, respectively; values in parentheses are standard errors.

Based on the results of the mediation mechanism analysis, it was found that the impact of the climate-adaptive city construction policy on corporate ESG scores is partially mediated by enhancing managerial environmental awareness. Specifically, the policy significantly increases managerial environmental awareness, which, in turn, has a significant positive effect on corporate ESG scores. At the same time, the direct impact of the policy on corporate ESG scores is also significant, indicating that managerial environmental awareness plays a partial mediating role between the policy and corporate ESG scores. Managerial environmental awareness is the key pathway through which the climate-adaptive city construction policy influences corporate ESG scores. Hypothesis H2 is validated, suggesting that the positive impact of the policy on corporate ESG scores is primarily achieved through directly improving managerial environmental awareness and indirectly promoting corporate sustainable development performance.

Additionally, considering the potential endogeneity between the key independent variable, climate-adaptive city construction, and the mediator variable, managerial environmental awareness, the results estimated from the mediation model may suffer from bias. To further enhance the credibility of the conclusions, we adopt the IV mediate model, following the approach of Dippel et al. (48), to test the causal relationship of the mediation effect. When there is endogeneity between the independent variable and the mediator, this method can separate the direct and mediation effects using a single instrument variable. Consistent with previous methods, the proximity to rivers are used as instrument variables. As shown in Table 6, the primary mediation effects are significant at the 1% level, and the explanatory power of the mediation effects is 82.57%. This indicates that, even after addressing the endogeneity issue, managerial environmental awareness remains an important channel for enhancing corporate ESG performance, and the mediation effect persists. In conclusion, the use of social networks measured by the primary interactions and local attitudes plays a mediating role in the influence of entrepreneurship on urban identity, thus validating Hypothesis H2.

**Table 6:** Causal relationship test of mediation effect: IV mediate model.

	ESG
	IV1
Mediation effect	1.118** (0.568)
Direct effect	0.236* (0.217)
Main effect	1.354*** (0.101)
Explanatory power of mediation effect	82.57%

***, **, and * indicate significance at the 1, 5, and 10% levels, respectively; values in parentheses are standard errors.

## 5 Main conclusions and managerial implications

### 5.1 Main conclusions

This study, based on data from Chinese listed companies from 2010 to 2021, employs difference-in-differences (DID) and text mining methods to examine the impact of climate adaptation policies on corporate ESG performance and their underlying mechanisms. The study finds the following: (1) Climate-adaptive city construction significantly improves overall corporate ESG performance. The study finds that the climate-adaptive city construction policy has a significant positive impact on companies’ environmental, social, and governance (ESG) performance, encouraging firms to enhance their sustainability efforts. This conclusion is supported by various robustness tests, demonstrating the effectiveness of the policy in improving overall ESG performance. (2) Managerial environmental awareness plays a key mediating role in the policy’s impact on corporate ESG performance. The results show that the climate-adaptive city construction policy enhances managerial attention to environmental issues, which in turn drives improvements in corporate ESG performance. Under policy pressure, managers place more emphasis on environmental responsibility, incorporating it into the company’s strategy and resource allocation, thereby improving the firm’s ability to address climate change. (3) The policy effects of climate-adaptive city construction exhibit strong robustness. After conducting several robustness tests, including combined fixed effects, placebo tests, and various endogeneity checks such as PSM-DID and instrumental variables (IV) methods, the study consistently finds the same results. The climate-adaptive policy continues to have a significant positive effect on corporate ESG performance under different testing conditions, showing the reliability and broad applicability of the policy’s impact.

### 5.2 Managerial implications

Based on the above research findings, this study provides the following managerial implications: (1) clarify policy guidance pathways and institutionalize managerial environmental awareness. Regulatory authorities can leverage instruments such as the Measures for the Administration of Corporate Environmental Information Disclosure or ESG reporting frameworks to mandate that boards of directors or senior executives incorporate sustainability and environmental topics into formal decision-making agendas. This would help institutionalize environmental concerns at the strategic level. Additionally, it is recommended that local environmental agencies and industry associations include environmental training for executives as part of their capacity-building initiatives. (2) Enhance incentive mechanisms to guide corporate ESG investment behavior. Fiscal instruments such as special government funds, tax deductions, and preferential green credit should be used to support enterprises undertaking energy-saving upgrades or green transitions within climate-resilient pilot zones. Furthermore, ESG performance could be integrated into local green finance evaluation systems to establish a measurable, market-based incentive structure. (3) Strengthen policy coordination to prevent distortion from overlapping instruments. Local governments are advised to consider existing policy tools—such as green credit guidelines and carbon trading schemes—when formulating supporting measures for climate-resilient city initiatives. This would help avoid redundant or conflicting signals that may undermine corporate commitment to ESG strategies. (4) Advance differentiated ESG indicator systems to offer tailored compliance pathways. Given the significant differences in capacity across firms of varying sizes and industries, regulators and industry bodies should jointly develop simplified ESG assessment models for small and medium-sized enterprises (SMEs), while promoting the creation of sector-specific ESG guidelines in key industries such as energy, manufacturing, and construction. These efforts would enhance the relevance and feasibility of ESG implementation.

### 5.3 Limitations and future directions

First, this study focuses on managerial environmental attention as a cognitive transmission mechanism and does not examine alternative pathways such as resource input or compliance responses. Future research may extend this framework by incorporating variables such as green investment and regulatory intensity, thereby constructing a more comprehensive multi-channel transmission model to further uncover how climate policies influence corporate ESG behavior in a holistic manner.

Second, due to limitations in data availability and consistency, this study focuses solely on A-share listed companies in China. However, listed firms generally have larger scales, more advanced governance structures, and more standardized information disclosure practices than small and medium-sized enterprises (SMEs). As a result, their responses to policy interventions may be more rapid, and the behavioral mechanisms may differ systematically. Therefore, the external validity of the study’s conclusions may be limited. Future research could expand the sample scope to include non-listed firms and SMEs across different regions or industries, in order to examine the applicability and heterogeneous effects of climate-resilient city policies in a broader corporate context. This would enhance the generalizability of the findings and the practical relevance of the policy implications.

Third, this study measures managerial environmental attention using the frequency of environment-related vocabulary in annual reports, a method that, while widely used in many studies, has its limitations. Firstly, the frequency of environmental terms in annual reports may be influenced by the company’s disclosure strategy, industry regulations, and other external factors, which may not fully reflect the true level of managerial environmental concern. Additionally, word frequency statistics measure the quantity of environmental content but do not consider its quality or depth, potentially leading to measurement errors. Furthermore, differences in disclosure practices, content, and emphasis across companies, industries, and years may introduce heterogeneity in the measurement. To address these limitations, future research could consider incorporating more diverse measurement methods, such as using executive behavior data (e.g., public speeches, media interviews) or survey-based approaches, to capture managerial environmental attention and attitudes more comprehensively. Additionally, combining qualitative analysis with text mining techniques could provide a more accurate understanding of the depth and quality of managerial attention to environmental issues in corporate annual reports.

Forth, due to data availability and consistency issues, this study has not fully incorporated all potential concurrent policies and their interaction effects. For instance, during the study period, China implemented several environmental and economic policies (such as carbon trading pilots and green credit initiatives), which may interact and influence corporate behavior. Although we address these concerns through IV-based robustness tests and placebo checks, data limitations prevent us from fully controlling for all concurrent policy effects. Future research could explore the use of more comprehensive policy databases and regional economic data, incorporating policy interaction indicators to more precisely assess the joint effects of climate-resilient city construction policies and other policies, thereby providing a better understanding of the net effect of these policies on corporate behavior.

Fifth, Improvements in corporate ESG performance often exhibit a time lag, and the impacts of climate-resilient city construction policies may not appear immediately. Instead, they may unfold in increasing, decreasing, or non-linear patterns over different time periods. However, due to limitations in manuscript length and data structure, this study does not employ an event-study specification to systematically capture the year-by-year effects following policy implementation. This may lead to an underestimation of the temporal heterogeneity of policy impacts. Therefore, we suggest that future research, conditional on data availability, adopt more flexible event-study frameworks to identify how policy effects evolve at different time points. Such approaches would enable a more comprehensive understanding of the medium- and long-term impacts of climate policies on corporate sustainable behavior.

## Data Availability

Publicly available datasets were analyzed in this study. This data can be found: company financial data were retrieved from Wind Financial Database. Access requires subscription at: https://www.wind.com.cn/.
