# Supplementary material for: The impact of climate-adaptive city construction pilot policies on corporate ESG performance
Source: Front Public Health. 2025 Sep 15;13:1646269. doi: 10.3389/fpubh.2025.1646269 (PMC12477152; doi:10.3389/fpubh.2025.1646269)
Supplement: Supplementary file 1 [file Table_1.docx]

Supplementary table 1 PSM—DID Test

|  | PSM—DID  (1) |
| --- | --- |
| Policy | 0.097***  (0.023) |
| Control Variables | Yes |
| R^2^ | 0.680 |
| Industry Fixed Effects | Yes |
| Year Fixed Effects | Yes |
| Cluster | Yes |

Note: ***, **, and * indicate significance at the 1%, 5%, and 10% levels, respectively; values in parentheses are standard errors.
